# Functional Divergence of *NOTCH1* and *NOTCH2* in Human Cerebral Organoids Reveals Receptor-Specific Roles in Early Corticogenesis

**DOI:** 10.3390/ijms26157309

**Published:** 2025-07-29

**Authors:** Sophia Yakovleva, Anastasia Knyazeva, Anastasia Yunusova, Elina Allayarova, Dmitriy Lanshakov, Anna Malashicheva, Tatiana Shnaider

**Affiliations:** 1 Institute of Cytology and Genetics, Siberian Branch of Russian Academy of Sciences, 630090 Novosibirsk, Russia; s.tschetschetkina@gmail.com (S.Y.); a.chvileva@g.nsu.ru (A.K.); e.allayarova@g.nsu.ru (E.A.); lanshakov@bionet.nsc.ru (D.L.); shnayder@bionet.nsc.ru (T.S.); 2 Department of Natural Sciences, V. Zelman Institute for Medicine and Psychology, Novosibirsk State University, 630090 Novosibirsk, Russia; 3 Institute of Cytology, Russian Academy of Sciences, 194064 Saint Petersburg, Russia; malashicheva@incras.ru

**Keywords:** cerebral organoids, Notch signaling pathway, neurodevelopment, cortex

## Abstract

The Notch signaling pathway is a critical regulator of embryonic brain development. Among its four mammalian receptors, Notch1 and Notch2 are particularly significant in the developing cortex, yet their roles in human neurodevelopment are not well understood. In murine cortex development, *Notch1* primarily regulates early progenitor identity and neurogenesis, while *Notch2* is required for maintaining radial glial cells at later stages. However, it is unclear whether these functions are conserved in the human developing brain. In this study, we used cerebral organoids as an in vitro model of early human corticogenesis and conducted lentiviral shRNA-mediated knockdowns of *NOTCH1* and *NOTCH2*. Our findings indicate that *NOTCH1* is essential for organoid growth, lumen morphogenesis, radial glial identity, and progenitor proliferation. In contrast, depleting *NOTCH2* did not significantly affect these early developmental processes. These results demonstrate that *NOTCH1* and *NOTCH2* have potentially non-redundant and temporally distinct roles in early human corticogenesis, reflecting receptor-specific specialization within the Notch signaling pathway.

## 1. Introduction

The Notch signaling pathway plays a vital role in early embryonic development by regulating cell fate determination, proliferation, and survival across various tissues. Its extensive pleiotropic effects are particularly critical in neurodevelopment [1,2,3]. Through direct cell–cell interactions, the Notch cascade maintains a finely tuned balance between the self-renewal of neural progenitor cells and their differentiation into neurons and various glial subtypes [4,5,6,7,8,9,10,11]. Additionally, it regulates apoptosis [12,13,14], neurite growth [15,16,17], axon arborization [18], and dendritic development [19]. Abnormal activity within this signaling cascade, often due to genetic variants in its core components, has been linked to neurodevelopmental disorders, including malformations of cortical development [20,21,22], intellectual disability [22], autism [22,23], bipolar disorder [24], and schizophrenia [25,26].

At the molecular level, the core of the Notch signaling cascade is represented by ligands and receptors, localized on adjacent cells. In mammals, including humans, four Notch receptors have been identified—Notch1, Notch2, Notch3, and Notch4. These receptors share a conserved structural architecture found across metazoan species [27], comprising an extracellular domain with epidermal growth factor (EGF)-like repeats, a single-pass transmembrane segment, and an intracellular domain responsible for transcriptional activation. Despite their structural similarity and partially overlapping expression profiles [28,29,30,31], these receptors demonstrate non-redundant roles during neurodevelopment. Among them, Notch1 [28,32,33,34] and Notch2 [12] are particularly crucial, whereas Notch3 [35] and Notch4 [36] have been shown to play more limited roles. This functional diversification is shaped by multifaceted aspects, including context-specific expression [37,38], different ligand-receptor combinations [39,40,41], and distinct downstream transcriptional outcomes [12,33,42].

During early neurodevelopment, both *Notch1* and *Notch2* are expressed in the telencephalon, particularly within the ventricular zone [29], indicating their crucial involvement in corticogenesis. Specifically, *Notch1* is pivotal in establishing radial glial cell (RGC) identity [34,43], whereas *Notch2* is not involved in this process. Both receptors are essential for the maintenance of RGCs, contributing to their proliferation [44], differentiation [45,46], and apoptosis [13,44]. Nevertheless, their temporal involvement differs: *Notch*1 primarily regulates early stages, while *Notch2* contributes to the later period [46].

Despite the highly functional conservation of the Notch signaling pathway among mammals, recent studies have revealed the emergence of human-specific “innovations” within this cascade. A striking example is the *NOTCH2NL* genes, human-specific *NOTCH2* paralogs expressed in RGCs, which are thought to promote the expansion of cortical progenitors and potentially contribute to the increase in human brain size [21,47,48,49]. In light of these findings, it is possible that canonical Notch receptors may display species-specific features during human neurodevelopment. Thus, while the receptor-specific functions of Notch1 and Notch2 have been well characterized in murine models, it remains unclear whether similar functional distinctions exist in human cortical development.

To determine the functions of *NOTCH1* and *NOTCH2* in early human corticogenesis, we employed the cerebral organoid (CO) system [50], an in vitro model that recapitulates key aspects of early human forebrain development. Using lentivirus-based shRNA-mediated depletion of *NOTCH1* and *NOTCH2,* we established their individual contributions to neural progenitor proliferation, tissue morphogenesis, and RGC specification. Our findings reveal receptor-specific specialization within the Notch signaling pathway that appears to be evolutionarily conserved across mammals, including humans. Furthermore, these results support the central role of Notch signaling in orchestrating early neurodevelopmental processes that shape the architecture of the human cerebral cortex.

## 2. Results

### 2.1. Depletion of NOTCH1, but Not NOTCH2, Alters External Morphology of COs

**Figure 1:**
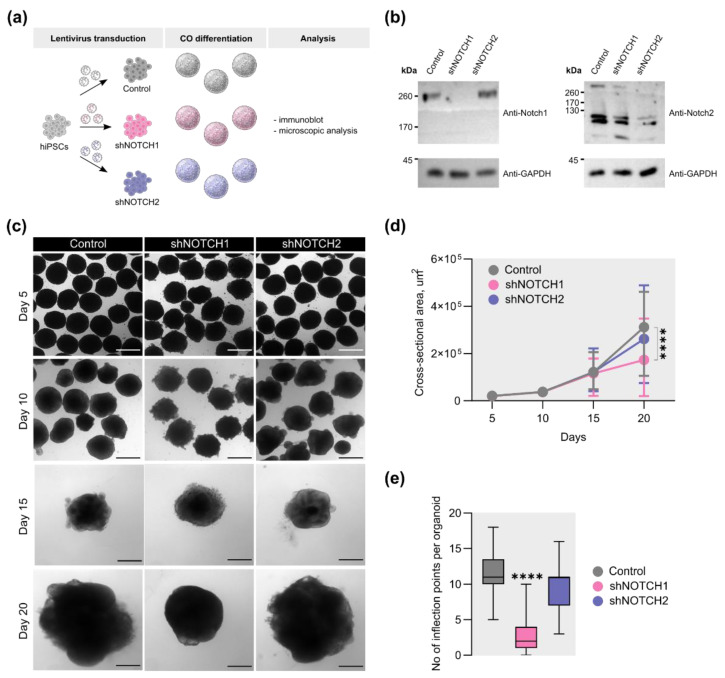
Effects of *NOTCH1* and *NOTCH2* knockdown on the size and external morphology of COs. (**a**) Scheme of experiment for lentivirus-mediated shRNA knockdown of *NOTCH1* and *NOTCH2*. The scheme was created using NIAID NIH BIOART Source: https://bioart.niaid.nih.gov/bioart/2182, https://bioart.niaid.nih.gov/bioart/399, and https://bioart.niaid.nih.gov/bioart/172 (accessed on 25 May 2025). (**b**) Representative western blot results of NOTCH1 and NOTCH2 protein in COs at 20 and 45 days of differentiation, respectively. GAPDH was used as a housekeeping control. (**c**) Representative brightfield image of COs on 5, 10, 15, 20 days of differentiation. The scale bar is 200 μm. (**d**) Quantitative analysis of the CO cross-sectional area on different days of the differentiation. Data points represent the median with minimum and maximum values. The Kruskal–Wallis test, followed by Dunn’s multiple comparisons test, was used for statistical analysis. ****—*p* < 0.0001; Day 5 *n* > 109 per group, Day 10 *n* = 102, Day 15 *n* > 73, Day 20 *n* > 92. (**e**) Quantitative analysis of the CO inflection points on day 20 of the differentiation. The box plot represents the median and quartiles with minimum and maximum values. The Kruskal–Wallis test, followed by Dunn’s multiple comparisons test, was used for statistical analysis. ****—*p* < 0.0001; *n* = 41 for each group.

To investigate the contributions of *NOTCH1* and *NOTCH2* to early human cortical development, we employed a selective knockdown approach using RNA interference in COs (Figure 1a). Due to the low efficiency of lentiviral transduction of COs (Figure S1), shRNAs targeting *NOTCH1* and *NOTCH2* were introduced at the hiPSC stage. Knockdown efficiency was validated by reduced levels of NOTCH1 and NOTCH2 protein in COs on days 20 and 45 of differentiation, respectively (Figure 1b and Figure S2).

It has been previously demonstrated that knockouts of *Notch1* and *Notch2* in mice result in severe embryonic developmental defects, including marked growth retardation [12,28,32]. Based on these findings, we first investigated whether knockdown of these receptors affects the growth of COs. CO size was assessed on differentiation days 5, 10, 15, and 20. By day 20, COs with decreased *NOTCH1* expression exhibited a significant reduction in cross-sectional area compared to the control group (Figure 1c). Quantitative analysis revealed a 1.8-fold decrease in size in the *NOTCH1* knockdown group (Figure 1d). In contrast, the knockdown of *NOTCH2* had no significant effect on CO size.

In addition to size reduction, *NOTCH1*-deficient COs displayed a notable simplification of surface morphology. To quantify changes in surface complexity, we assessed the number of inflection points [51]. On day 20, *NOTCH1*-deficient COs showed a 5.5-fold decrease in inflection points compared to control (Figure 1e), whereas depletion of *NOTCH2* did not affect this parameter. These results highlight a specific requirement for *NOTCH1*, but not *NOTCH2*, in the early expansion and structural organization of human COs.

### 2.2. Differential Effects of NOTCH1 and NOTCH2 on Apical Polarity and Ventricle-Like Structures

Previous studies have demonstrated that the Notch signaling pathway drives morphogenesis during embryonic development [52], including neurodevelopment [53]. One of the hallmark events in early brain morphogenesis is the formation of the ventricular system from the neural tube lumen. Similarly, ventricle-like structures (VLSs) also emerge during CO differentiation. To assess the role of *NOTCH1* and *NOTCH2* in VLS development, we analyzed the number, area, and thickness of VLSs upon knockdown of each receptor.

**Figure 2:**
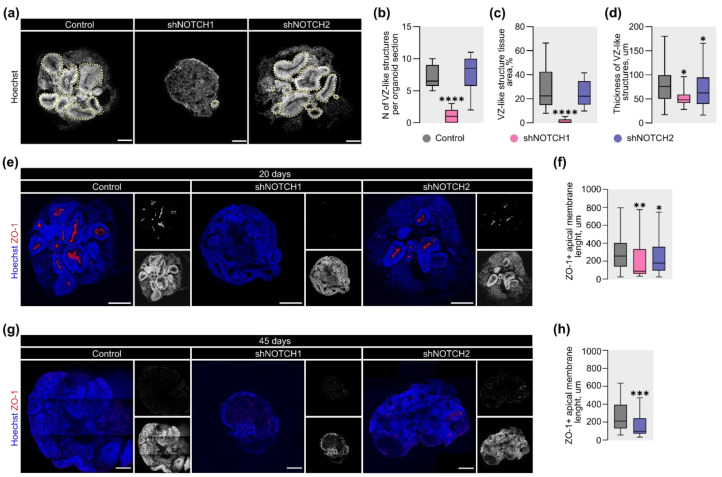
*NOTCH1* regulates ventricle-like structure formation and apical polarity in COs. (**a**) Representative fluorescent images of the entire organization of COs at 20 days of differentiation. The yellow dotted line labels VLSs. The scale bar is 250 μm. (**b**–**d**) Quantitative analysis of morphological parameters of COs: (**b**) the number of VLSs per CO section, (**c**) VLS tissue area per CO section, (**d**) thickness of VLSs at 20 days of differentiation. The box plot represents the median and quartiles with minimum and maximum values. The Kruskal–Wallis test, followed by Dunn’s multiple comparisons test, was used for statistical analysis. ****—*p* < 0.0001; *—*p* < 0.02; *n* > 15 for each group. (**e**,**g**) Representative fluorescent images of apical membranes labeled by ZO-1 (red) at 20 and 45 days of differentiation, respectively. Separated channels are presented in grayscale. The scale bar is 250 μm. (**f**) Quantitative analysis of the perimeter of apical membrane in COs at 20 days of differentiation. The box plot represents the median and quartiles with minimum and maximum values. The Kruskal–Wallis test, followed by Dunn’s multiple comparisons test, was used for statistical analysis. **—*p* < 0.001; *—*p* < 0.02; *n* > 29 for each group. (**h**) Quantitative analysis of the perimeter of apical membrane in COs at 45 days of differentiation. The box plot represents the median and quartiles with minimum and maximum values. The Mann–Whitney test was used for statistical analysis. ***—*p* < 0.0005; *n* > 44 for each group.

By day 20, COs with reduced *NOTCH1* expression exhibited severe defects in lumenization (Figure 2a). Quantitative analysis revealed a 6.5-fold reduction in the number of VLSs per CO section (Figure 2b) and a 38.3-fold decrease in their total area compared to the control group (Figure 2c). In contrast, the number and area of VLSs in *NOTCH2*-depleted COs remained comparable to the control group. However, analysis of VLS thickness showed a moderate reduction in both knockdown conditions: 1.6-fold in *NOTCH1*-deficient COs and 1.2-fold in those lacking *NOTCH2* (Figure 2d).

To examine the basis of these defects, we analyzed the apical polarity, which plays a pivotal role in lumen formation. Using ZO-1 as a tight junction marker, we found that the apical membrane perimeter of VLSs was markedly reduced in *NOTCH1*-depleted COs at day 20 (Figure 2e), with a 2.9-fold decrease relative to the control group (Figure 2f). A more modest 1.4-fold reduction of this parameter was also observed in the *NOTCH2* knockdown group.

To investigate whether these early defects persist at later stages, we assessed the ZO-1+ apical perimeter at day 45 (Figure 2g). Due to the scarcity of identifiable VLSs in the *NOTCH1*-deficient group, these COs were excluded from the analysis. In contrast, *NOTCH2*-deficient COs retained a smaller ZO-1+ apical perimeter of VLSs at this stage, with a 2.2-fold reduction compared to controls (Figure 2h).

Together, these results indicate that *NOTCH1* is essential for initiating and maintaining apical-basal polarity and VLS formation in COs. The relatively mild defects observed upon *NOTCH2* depletion likely indicate a more limited and non-redundant role for this receptor in early human cortical morphogenesis.

### 2.3. NOTCH1 Is Required for RGC Fate Specification and Maintenance of Cortical Progenitor Identity

During early development, tissue morphogenesis is closely linked to cell fate specification [54]. Disruptions in this process in embryonic mouse brain are primarily determined by changes in the activity of the Notch signaling pathway [7,55]. Moreover, previous studies have shown a direct link between *Notch1* and RGC fate determination in developing mouse cortex [34]. To investigate whether *NOTCH1* and *NOTCH2* contribute similarly in humans, we examined the expression of the RGC marker PAX6 in COs.

**Figure 3:**
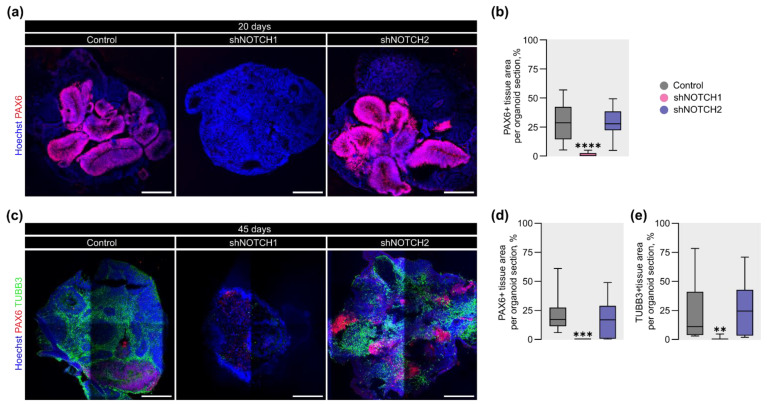
Depletion of *NOTCH1* disrupts RGC fate commitment and neurogenesis in COs. (**a**) Representative fluorescent images of PAX6 expression in COs at 20 days of differentiation. PAX6 (red) was used as a marker of RGC. The scale bar is 250 μm. (**b**) Quantitative analysis of PAX6+ tissue area per CO section at 20 days of differentiation. The box plot represents the median and quartiles with minimum and maximum values. The Kruskal–Wallis test, followed by Dunn’s multiple comparisons test, was used for statistical analysis. ****—*p* < 0.0001; *n* > 28 for each group. (**c**) Representative fluorescent images of PAX6 and TUBB3 expression in COs at 45 days of differentiation. PAX6 was used as a marker of RGC. TUBB3 (green) was used as a marker of neurons. The scale bar is 250 μm. (**d**,**e**) Quantitative analysis of PAX6+ or TUBB3+ tissue area per CO section at 45 days of differentiation, respectively. The box plot represents the median and quartiles with minimum and maximum values. The Kruskal–Wallis test, followed by Dunn’s multiple comparisons test, was used for statistical analysis. **—*p* = 0.0017; ***—*p* < 0.0002; *n* > 9 for each group.

Immunofluorescence analysis at day 20 revealed an almost complete absence of PAX6+ RGCs in *NOTCH1*-depleted COs, while *NOTCH2* knockdown did not affect PAX6 expression (Figure 3a). Quantitative analysis confirmed a 28.6-fold reduction in PAX6+ area relative to total CO area in *NOTCH1*-deficient COs compared to controls (Figure 3b).

To assess whether this phenotype persisted at later stages, we analyzed PAX6 expression at day 45 (Figure 3c). Similar to observations at day 20, PAX6+ regions were virtually absent in COs with *NOTCH1* knockdown, indicating its sustained effect on RGC identity. (Figure 3d). In contrast, *NOTCH2*-deficient COs showed normal PAX6 expression comparable to controls. Furthermore, we examined neuronal differentiation using the marker TUBB3. The TUBB3+ area was nearly absent in the *NOTCH1*-depleted group at day 45 (Figure 3e), suggesting that early disruption of progenitor identity led to impaired neurogenesis at later stages.

These findings underscore the crucial function of *NOTCH1* in establishing cell identity and sustaining the RGC pool during early human brain development. In contrast to *NOTCH1*, *NOTCH2* appears to play only a modest or non-essential role in regulating RGC identity and subsequent neurogenic progression.

### 2.4. NOTCH1 Regulates the Proliferative Capacity and Division Mode of RGC in COs

**Figure 4:**
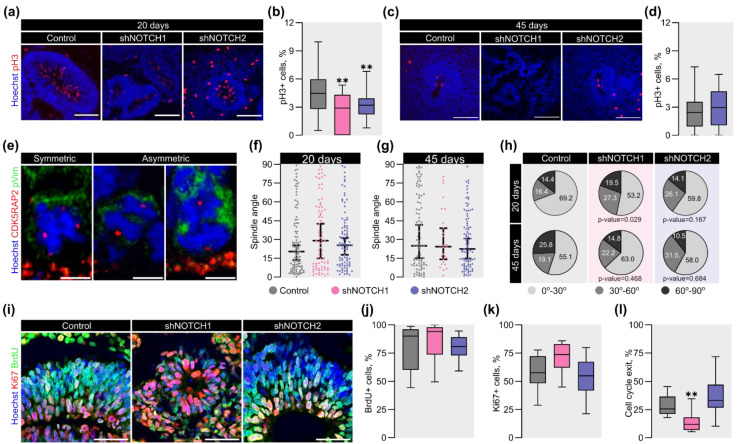
*NOTCH1* regulates proliferation, spindle orientation, and cell cycle dynamics in RGCs of COs. (**a**) Representative confocal images of pH3 (red) staining in VLS at 20 days of differentiation. pH3 marks mitotic cells. The scale bar is 100 μm. (**b**) Quantitative analysis of percentage pH3+ cells in VLS at 20 days of differentiation. The box plot represents the median and quartiles with minimum and maximum values. The Kruskal–Wallis test, followed by Dunn’s multiple comparisons test, was used for statistical analysis. **—*p* < 0.001; *n* > 18 for each group. (**c**) Representative confocal images of pH3 expression in VLS at 45 days of differentiation. VLS were absent in COs with depletion of *NOTCH1*. Thus, they were excluded from the analysis. The scale bar is 100 μm. (**d**) Quantitative analysis of percentage pH3+ cells in VLS at 45 days of differentiation. The box plot represents the median and quartiles with minimum and maximum values. The Mann–Whitney test was used for statistical analysis. *n* > 23 for each group. (**e**) Representative confocal images of the mitotic spindle orientation. CDK5RAP2 (red) and pVim (green) were used as a centriole and dividing cell markers, respectively. The scale bar is 5 μm. (**f**,**g**) Quantitative analysis of mitotic spindle angle at 20, 45 days of differentiation, respectively. The dot plot represents the distribution of values with the median and a 95% confidence interval. The Kruskal–Wallis test, followed by Dunn’s multiple comparisons test, was used for statistical analysis. *n* > 77 for each group at 20 days, *n* > 27 for each group at 45 days. (**h**) Quantitative analysis of the distribution of cell divisions at 20, 45 days of differentiation. Pie charts represent the distribution of horizontal (0–30°), oblique (30–60°) and vertical (60–90°) orientations of division spindles. The chi-square test was used for statistical analysis. (**i**) Representative confocal images of BrdU (green) and Ki67 (red) staining at 20 days of differentiation. The scale bar is 50 μm. (**j**–**l**) Quantitative analysis of cell cycle parameters at 20 days of differentiation. (**j**) S-phase cells (BrdU+/total cells); (**k**) proliferative index (Ki67+/total cells); (**l**) cell cycle exit (BrdU+Ki67−/BrdU+cells). The box plot represents the median and quartiles with minimum and maximum values. The Kruskal–Wallis test, followed by Dunn’s multiple comparisons test, was used for statistical analysis. **—*p* < 0.001; *n* > 12 for each group.

After acquiring RGC identity, their regulated proliferation is a critical next step in cortical development. Previous studies have shown that murine Notch receptors regulate neuronal cell proliferation [46,56,57,58,59]. To assess whether *NOTCH1* and *NOTCH2* influence RGC division during human cortical development, we quantified mitotic activity using phospho-histone H3 (PH3) immunostaining of COs at day 20 of differentiation (Figure 4a). Compared to controls, COs with reduced *NOTCH1* expression exhibited a 1.5-fold decrease in VLS-located PH3+ cells, while *NOTCH2*-depleted COs showed a 1.4-fold reduction (Figure 4b). On day 45, COs lacking *NOTCH1* were excluded from the analysis due to insufficient VLS formation (Figure 4c). In contrast, *NOTCH2*-depleted COs displayed no statistically significant difference in PH3+ cells compared to controls, suggesting a milder effect on proliferative capacity (Figure 4d).

While reduced mitotic activity suggests impaired proliferation at 20 days of differentiation, changes in the orientation of cell division may further inform how progenitor dynamics are altered upon receptor depletion. Given the role of Notch signaling in regulating RGC division patterns [60,61], we analyzed mitotic spindle orientation as a suggestive, albeit indirect, indicator of division mode. Using CDK5RAP2 immunostaining to label centrioles (Figure 4e), we measured the spindle angles in mitotic RGCs at days 20 and 45 of CO differentiation in RGCs at days 20 and 45 of CO differentiation (Figure 4f,g). Although the median spindle angles did not differ significantly between groups, the analysis of the distribution of spindle angles revealed a shift in division geometry. On day 20, NOTCH1-depleted COs showed a significant reduction (16%) in the proportion of cells undergoing horizontal divisions (0–30°) (Figure 4h). By day 45, this difference was not observed, suggesting a stage-specific effect. In contrast, the *NOTCH2* knockdown did not significantly alter the spindle angle distribution at either time point.

While changes in spindle orientation have often been interpreted as a proxy for symmetric versus asymmetric divisions, it is important to note that this relationship is not absolute and remains debated. Therefore, we complemented this analysis by assessing cell cycle dynamics using BrdU and Ki-67 labeling to estimate S-phase entry and overall proliferation. The BrdU^+^/Ki-67^−^ fraction was used to approximate cell cycle exit. BrdU and Ki-67 labeling provided estimates of S-phase and proliferative cell fractions, respectively. Together, these measures enabled a more comprehensive evaluation of proliferative status across experimental conditions. BrdU and Ki67 labeling showed no significant differences across experimental groups, indicating comparable rates of cell cycle entry and proliferation. (Figure 4i–k). However, *NOTCH1*-deficient COs exhibited a 2.1-fold reduction in cell cycle exit compared to controls (Figure 4l), whereas *NOTCH2* knockdown had no significant effect. Interestingly, although an increase in vertical spindle orientation is typically associated with neurogenic asymmetric divisions, this was not accompanied by enhanced cell cycle exit in our model. This dissociation suggests a disrupted coupling between division geometry and fate progression, potentially reflecting premature progenitor depletion or destabilization of RGC identity, in line with the observed reduction in PAX6 expression.

Together, these results underscore the pivotal role of *NOTCH1* in sustaining RGC proliferation, regulating division mode, and maintaining the proliferative output required for proper cortical development. In contrast, *NOTCH2* has a more modest and possibly non-redundant contribution to these processes.

## 3. Discussion

Our results suggest that *NOTCH1* and *NOTCH2* receptors undergo functional divergence during early human cortical development. Through selective knockdown of *NOTCH1* and *NOTCH2* in COs, we demonstrated that *NOTCH1* is essential for establishing cortical tissue architecture, RGC specification, and promoting progenitor proliferation. By contrast, *NOTCH2* exerted a significantly weaker influence on the same developmental processes in the CO system.

The Notch signaling pathway is crucial in mammalian embryonic development, regulating tissue growth and morphogenesis. Previous studies in mice have shown that knockout of *Notch1* or *Notch2* results in severe developmental abnormalities, including pronounced growth retardation and disrupted organogenesis [12,28,32]. Consistent with these findings, we observed that *NOTCH1*, but not *NOTCH2*, was required for the normal growth and structural complexity of COs. *NOTCH1*-depleted COs showed marked delay and impaired surface folding, accompanied by severe defects in VLS formation. In contrast, despite the established importance of *Notch2* in murine embryogenesis, *NOTCH2* depletion resulted in relatively mild effects on CO growth and morphogenesis, suggesting *NOTCH2* plays a more limited or context-dependent role, possibly acting in later developmental windows. Overall, while our current observations suggest that *NOTCH2* may have a weaker impact during early cortical development, we acknowledge that additional experiments are required to clarify potential non-redundancy or cooperation between *NOTCH1* and *NOTCH2*, such as rescue approaches or combined knockdowns.

Since proper apicobasal polarity is essential for cortical tissue organization, we assessed whether the impaired morphogenesis in *NOTCH1*-deficient COs was linked to defects in cell polarity. Consistent with this hypothesis, we found a marked disruption of apical membrane architecture upon *NOTCH1* depletion. These findings align with prior studies implicating Notch signaling in tissue morphogenesis and epithelial polarity [53,55,62,63], and extend them by demonstrating a dominant function for *NOTCH1*, relative to *NOTCH2*, in driving apical morphogenesis in human cortical-like tissues. Although *NOTCH2* knockdown modestly reduced apical perimeter, this effect was weaker and only apparent later, suggesting a subsidiary or compensatory role.

Tissue morphogenesis and cell fate specification are closely coupled during early development. In our model, disruption of apical polarity and lumenization in *NOTCH1*-deficient COs was accompanied by a reduction in the population of PAX6-positive cells, indicating that *NOTCH1* is required for establishing and maintaining progenitor identity. These findings are consistent with the pivotal role of *Notch1* in regulating RGC fate in the developing mouse cortex [34]. On day 45, we observed significantly reduced areas positive for the postmitotic neuronal marker TUBB3, suggesting a global developmental delay in *NOTCH1*-deficient COs caused by premature depletion of the progenitor pool. Collectively, these results are consistent with previous observations that apical polarity and cell fate determination are interrelated processes in cerebral cortex development [64] and could be regulated by *NOTCH1*, but not *NOTCH2*.

Additionally, our findings reveal a complex and nonlinear relationship between cell cycle dynamics and cell fate determination under *NOTCH1* depletion. At 20 days of differentiation, *NOTCH1*-deficient COs exhibited a decreased proportion of horizontally dividing RGCs, which was paradoxically accompanied by a marked reduction in cell cycle exit. Although a direct correlation between mitotic spindle orientation and the asymmetric outcome of neural progenitor divisions in mammals remains debated [65,66,67], several studies support the notion that spindle orientation can influence the fate of daughter cells through Notch signaling [45,60,68,69,70,71]. However, in our model, this coupling appears to be disrupted. In line with a previous study [45], which demonstrated that Notch signaling is required to maintain RGC identity during both symmetric and asymmetric divisions, our findings suggest that in the absence of *NOTCH1*, progenitors may initiate asymmetric divisions morphologically but fail to complete neurogenic programs. Loss of apicobasal polarity, reduced PAX6 expression, and impaired epithelial organization in *NOTCH1*-deficient COs suggest that progenitors lose the structural and molecular context necessary to execute neurogenic programs, even when division geometry would otherwise support it.

In summary, our findings suggest that *NOTCH1* and *NOTCH2* have unique, temporally distinct roles during early human cortical development. These results not only highlight the receptor-specific functions within the Notch pathway but also provide insights into how disruptions in this pathway may contribute to certain neurodevelopmental disorders. Moreover, our study emphasizes the value of COs as a human-specific model for exploring the spatial and temporal complexities of developmental signaling pathways. However, brain organoid models have several significant limitations that should be considered when interpreting the results. These include the absence of vascular and immune cells, limited maturation, impaired regional patterning, and reduced spatial organization. Organoids also exhibit batch variability, which can impact the reproducibility of experiments and, consequently, affect the conclusions drawn from them. Recent advances are likely to address some of these issues, enhancing our understanding of various processes and adding value to the obtained data. Nevertheless, at the current moment, COs remain a physiologically relevant model for studying various aspects of early human cortical development, including Notch signaling activities. Future research should also focus on identifying the receptor-specific downstream targets of NOTCH1 and NOTCH2, as well as examining how external factors influence their activity in the developing human cortex.

## 4. Materials and Methods

### 4.1. Cell Cultures

The hiPSC line iTAF1-36 was obtained from a healthy donor and has been characterized previously [72]. hiPSCs were maintained on plates coated with Corning^®^ Matrigel^®^ hESC-Qualified Matrix (354277, Corning Life Sciences, Corning, NY, USA) in complete mTeSR™1 medium (85850, STEMCELL Technologies, Vancouver, BC, Canada) and cultured at 37 °C in an incubator with 5% CO_2_. The passaging of hiPSCs was performed using StemPro™ Accutase™ Cell Dissociation Reagent (A1110501, Thermo Fisher Scientific, Waltham, MA, USA) every 4–5 days.

HEK293T (Phoenix) cells were maintained on plates coated with 0.1%gelatin solution (G1890-100G, Sigma-Aldrich, St. Louis, MO, USA) in growth medium (10% Fetal Bovine Serum (Thermo Fisher Scientific, A5670701, USA), 87% DMEM high glucose (Wuhan Servicebio Technology Co., G4511-500ML, Wuhan, China), 1× Pen/Strep (bn-3E1B, BioinnLabs, Rostov-on-Don, Russia), 1 mM L-glutamine (GLN-B, Capricorn Scientific, Ebsdorfergrund, Germany), and 1× MEM NEAA (NEAA-B, Capricorn Scientific, Ebsdorfergrund, Germany)) and cultured at 37 °C in an incubator with 5% CO_2_. Cell passaging was performed using 0.25% Trypsin (bn-3D1C, BioinnLabs, Rostov-on-Don, Russia) once a week.

### 4.2. Production of Second-Generation Lentiviruses and Transduction

HEK293T cells were seeded the day before transfection at a density of 8 × 10^4^ cells/cm^2^ in culture dishes. The next day, cells were co-transfected with shRNA-expressing plasmids (pLKO-shNOTCH1 and pLKO-shNOTCH2 [73], or pLKO.1-TRC [74] (10879, Addgene, Watertown, MA, USA), the packaging plasmid psPAX2 (12260, Addgene, Watertown, MA, USA), and the envelope plasmid pMD2.G (12259, Addgene, Watertown, MA, USA) at a ratio of 4:2:1. The pLKO.1-TRC plasmid containing a non-hairpin insert was used as a negative control. Transfection was carried out using polyethylenimine (408727, Sigma-Aldrich, St. Louis, MO, USA) at the ratio of 2 μg PEI per 1 μg DNA. Cells were incubated overnight with the transfection mix. The next day, the medium was replaced with fresh medium. Viral supernatants were collected 24 h later, filtered through a 0.45 µm syringe filter to remove cellular debris, and used immediately for transduction or stored at −80 °C.

hiPSCs were plated in a 6-well plate at a density of 2.5 × 10^4^ cells/cm^2^. The following day, cells were transduced with lentiviruses. The following day, cells were transduced with the prepared second-generation lentiviruses in the presence of 10 μg/mL Polybrene^®^ (41122100, Sigma-Aldrich, St. Louis, MO, USA) to enhance transduction efficiency. After 24 h of incubation, the medium was replaced with complete mTeSR^TM^1 medium. Cells were cultured under standard conditions until they reached approximately 90% confluency prior to further processing.

### 4.3. Generation of COs

COs were differentiated from lentiviral transduced hiPSCs according to the previously described protocol with minor modifications [50]. A suspension containing 3 × 10^6^ cells was seeded into the AggreWell™800 plate (34811, STEMCELL Technologies, Vancouver, BC, Canada), coated with Anti-Adherence Rinsing Solution (07010, STEMCELL Technologies, Vancouver, BC, Canada). To improve cell survival, 20 μM Y27632 (72304, STEMCELL Technologies, Vancouver, BC, Canada) was added to the medium for embryoid bodies (EBs) (80% DMEM/F12 (11320033, Thermo Fisher Scientific, Waltham, MA, USA), 20% KnockOut™ Serum Replacement (10828028, Thermo Fisher Scientific, Waltham, MA, USA), 1 mM L-glutamine, 1× MEM NEAA, 0.1 mM 2-mercaptoethanol (Sigma-Aldrich, 805740, USA), 4 ng/mL bFGF (13256-029, Thermo Fisher Scientific, Waltham, MA, USA), and 1× Pen/Strep). On day 2, EBs were transferred to a 6-well plate coated with Anti-Adherence Rinsing Solution, and half of the medium was replaced daily. On day 5, the medium was replaced with EB medium without bFGF. On day 7, the medium was changed to neural induction medium (96% DMEM/F12, 1× N-2 Supplement (17502048, Thermo Fisher Scientific, Waltham, MA, USA), 1 mM L-glutamine, 1× MEM NEAA, 0.1 mM 2-mercaptoethanol, 1× Pen/Strep, and 1 μg/mL heparin (PHR8927, Sigma-Aldrich, St. Louis, MO, USA)). On day 11, COs were embedded in 10 μL Corning^®^ Matrigel^®^ Growth Factor Reduced Basement Membrane Matrix (354230, Corning Life Sciences, Corning, NY USA). The COs were cultivated in a medium for CO differentiation without vitamin A (50% DMEM/F12, 50% Neurobasal^TM^ medium (21103049, Thermo Fisher Scientific, Waltham, MA, USA), 0.5× N-2 Supplement, 1× B-27^TM^ Supplement minus vitamin A (12587010, Thermo Fisher Scientific, Waltham, MA, USA), 1 mM L-glutamine, 1× MEM NEAA, 0.1 mM 2-mercaptoethanol, 1× Pen/Strep, and 2.5 μg/mL insulin (I9278, Sigma-Aldrich, St. Louis, MO, USA)) in 100 mm dishes, pretreated with Anti-Adherence Rinsing Solution. On day 15, COs were removed from the Matrigel^®^ Matrix and transferred into a CO differentiation medium with vitamin A (50% DMEM/F12, 50% Neurobasal^TM^ medium, 0.5× N-2 Supplement, 1× B-27^TM^ Supplement (17504044, Thermo Fisher Scientific, Waltham, MA, USA), 1 mM L-glutamine, 1× MEM NEAA, 0.1 mM 2-mercaptoethanol, 1× Pen/Strep, 2.5 μg/mL insulin, 0.8 mM ascorbic acid (013-12061, FUJIFILM Wako Pure Chemical Corporation, Osaka, Japan), and 20 mM HEPES (HEP-B, Capricorn Scientific, Ebsdorfergrund, Germany)). COs were cultivated at 80 rpm in the CO_2_ Incubator Shaker (CS315, Radobio Scientific Co., Ltd., Shanghai, China) for continuous agitation. The medium was changed every 4 days.

The COs were treated with 10 μM BrdU for 24 h for BrdU labeling.

### 4.4. Fixation and Cryosectioning

COs were washed with PBS and fixed in 4% paraformaldehyde (141451.1211, AppliChem, Darmstadt, Germany) for 1 h at RT. Then, samples were dehydrated in a 30% sucrose (SA00201000, Medigene, Novosibirsk, Russia) solution in PBS at 4 °C overnight. Samples were subsequently embedded in a 7.5% gelatin (G1890, Sigma-Aldrich, St. Louis, MO, USA)/10% sucrose solution and frozen in molds using liquid nitrogen.

Cryosectioning was performed on an HM 550 Cryostat (Thermo Fisher Scientific, Waltham, MA, USA) with Epredia™ Ultra Disposable Microtome Blades (3053835, Epredia™, Kalamazoo, MA, USA). Slices of 30 μm thickness were collected on Epredia™ SuperFrost Plus™ adhesion microscope slides (11300, Epredia™, Kalamazoo, MA USA), air-dried overnight at RT, and stored at −20 °C until further use.

### 4.5. Immunohistochemistry and Imaging

Slides were washed with PBS and incubated with primary antibodies diluted in a blocking buffer (5% FBS, 0.2% Triton™ X-100 (11436631, Thermo Fisher Scientific, Waltham, MA, USA), and 2% BSA (A3311, Sigma-Aldrich, St. Louis, MO, USA)) overnight at RT on an orbital shaker at 50 rpm. After three washes with PBS, sections were incubated with secondary antibodies and Hoechst 33258 (14530, Sigma-Aldrich, St. Louis, MO, USA) diluted in PBS for 2 h at RT. The list of primary and secondary antibodies is presented in Table S1.

For BrdU staining, an additional DNA denaturation step was performed before incubation with primary antibodies. The slides were treated with 1.2 M HCl at 37 °C for 30 min, followed by neutralization in a 50 mM borate buffer at 37 °C for 30 min.

Fluorescent images were acquired using a confocal microscope Fluoview FV3000 (Olympus, Tokyo, Japan), and LSM 510 META (Zeiss, Oberkochen, Germany). During COs differentiation, phase-contrast images were taken using an Axio Observer Z1 microscope (Zeiss, Oberkochen, Germany).

### 4.6. Image Processing and Analysis

Fluorescent image analysis was performed using ImageJ software (version 1.54p) [75]. Tissue area parameters, ZO-1 length, and thickness of VLS were quantified with the “Freehand selection” tool. The area of the VLSs was considered as the difference between the areas bounded by the basal and apical membranes. VLS thickness was measured according to a previous publication [76]. The percentage of PH3+, BrdU+, and Ki67+ cells was quantified using the “Cell counter” plugin. Mitotic spindle angles were measured with the angle tool.

Quantitative analysis of brightfield images was performed using a custom Python-based script with the OpenCV (cv2) library (version 3.7.0). First, input images were converted to grayscale using the cv2.cvtColor function. Binary thresholding was then applied using Otsu’s method (cv2.threshold) to generate black-and-white images suitable for further segmentation.

To enhance the separation of adjacent or overlapping COs, morphological operations were performed. Specifically, morphological closing (cv2.morphologyEx) and dilation (cv2.dilate) were applied to refine the boundaries between white and black regions. To further delineate individual COs, a distance transform (cv2.distanceTransform) was employed to calculate the Euclidean distance between each non-zero pixel and the nearest zero-valued pixel. The processed binary images were then analyzed using the connected components labeling algorithm (cv2.connectedComponentsWithStats) to identify discrete objects representing individual COs. The CO cross-sectional area was calculated for each detected component, and contours were visualized to validate segmentation accuracy.

Inflection points were measured according to the previous publication [51].

### 4.7. Western Blotting

COs were washed with PBS and homogenized in ice-cold RIPA buffer supplemented with 5 μM NaF (S2002, Sigma-Aldrich, St. Louis, MO, USA), 1× PhosSTOP™ (4906845001, Roche, Basel, Sweden), and 1× cOmplete™ Protease Inhibitor Cocktail (Roche, 11836170001, Sweden). Protein concentrations were determined by Pierce™ BCA Protein Assay Kits (23225, Thermo Fisher Scientific, Waltham, MA, USA). Equal amounts of total protein (20 μg) were loaded per lane and separated in a 10% SDS-polyacrylamide gel at 90 V and 400 mA for 1.5–2 h. Then, proteins were transferred to Immun-Blot^®^ Low Fluorescence PVDF Membrane (1620264, Bio-Rad, Hercules, CA, USA) using a transfer buffer with methanol at 90 V and 350 mA for 2 h. Membranes were blocked using 5% non-fat milk (68514-61-4, NeoFroxx, Einhausen, Germany) for 1 h at RT and incubated with primary antibodies (Table S1) in 5% non-fat milk overnight at 4 °C on a roller shaker. After three washes in the TBST buffer, membranes were incubated with HRP-conjugated secondary antibodies for 2 h at RT. Further, the membrane was washed with TBST for 15 min and then visualized by Clarity™ Western ECL Substrate (1705060, Bio-Rad, Hercules, CA, USA) and detected in the iBright™ FL1000 Imaging System (Thermo Fisher Scientific, Waltham, MA, USA).

Membranes were stripped according to a previously published protocol [77]. Briefly, membranes were washed with distilled water and incubated in stripping buffer (20 mM Tris-HCl pH 7.5 (8796-300, Sigma-Aldrich, St. Louis, MO, USA), 6 M guanidine hydrochloride (14000295.5000, Diaem, Moscow, Russia), 0.2% NP-40 (A1112.0500, Applichem, Darmstadt, Germany), 0.1 M β-mercaptoethanol, and deionized water up to 100 mL) three times for 10–15 min at RT on an orbital shaker, followed by three 15-min washes with TBST buffer. Next, membranes were re-blocked in 5% non-fat milk and reused for subsequent antibody incubations. Image processing and analysis were performed using iBright Analysis Software (version 5.4.0) (Thermo Fisher Scientific, Waltham, MA, USA).

### 4.8. Statistical Analysis

The raw data are provided in Table S2. The sample sizes are indicated in the figure legends. The Shapiro–Wilk test was used to assess data normality. Statistical hypotheses were tested using the Kruskal–Wallis test. Dunn’s test corrected for multiple comparisons.

## Data Availability

Data are contained within the article and Supplementary Materials.

## Supplementary Materials

The following supporting information can be downloaded at: https://www.mdpi.com/article/10.3390/ijms26157309/s1.

## Abbreviations

The following abbreviations are used in this manuscript:

bFGF	basic Fibroblast Growth Factor
BrdU	Directory of open access journals
CO	Cerebral organoid
EB	Embryoid body
hiPSC	Human-induced pluripotent stem cell
shRNA	Short-hairpin RNA
VLS	Ventricle-like structure
