# Supplementary material for: Functional Divergence of NOTCH1 and NOTCH2 in Human Cerebral Organoids Reveals Receptor-Specific Roles in Early Corticogenesis
Source: Int J Mol Sci. 2025 Jul 29;26(15):7309. doi: 10.3390/ijms26157309 (PMC12347189; doi:10.3390/ijms26157309)
Supplement: Supplementary file 1 [file ijms-26-07309-s001.zip › Figure S1.pdf]

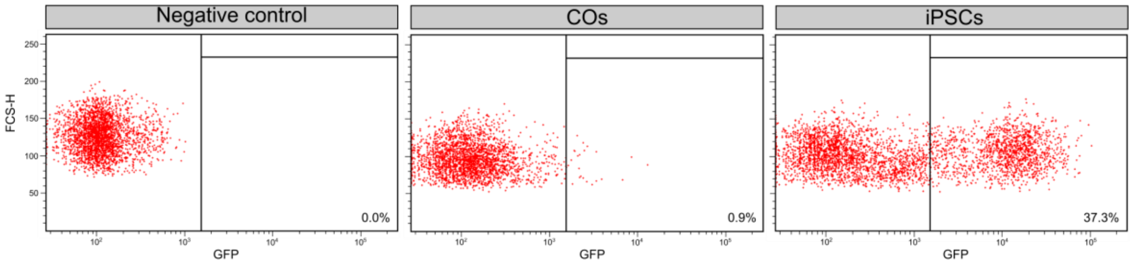

Figure S1. Validation of efficiency GFP-lentivirus transduction of hiPSC and COs.

Flow cytometry quantification of the GFP+ fraction of iPSCs and COs, transduced with CMV-GFP. The percentage of GFP^+ cells is indicated in the lower right corner of each plot.
