# Supplementary material for: Functional Divergence of NOTCH1 and NOTCH2 in Human Cerebral Organoids Reveals Receptor-Specific Roles in Early Corticogenesis
Source: Int J Mol Sci. 2025 Jul 29;26(15):7309. doi: 10.3390/ijms26157309 (PMC12347189; doi:10.3390/ijms26157309)
Supplement: Supplementary file 1 [file ijms-26-07309-s001.zip › Figure S2.pdf]

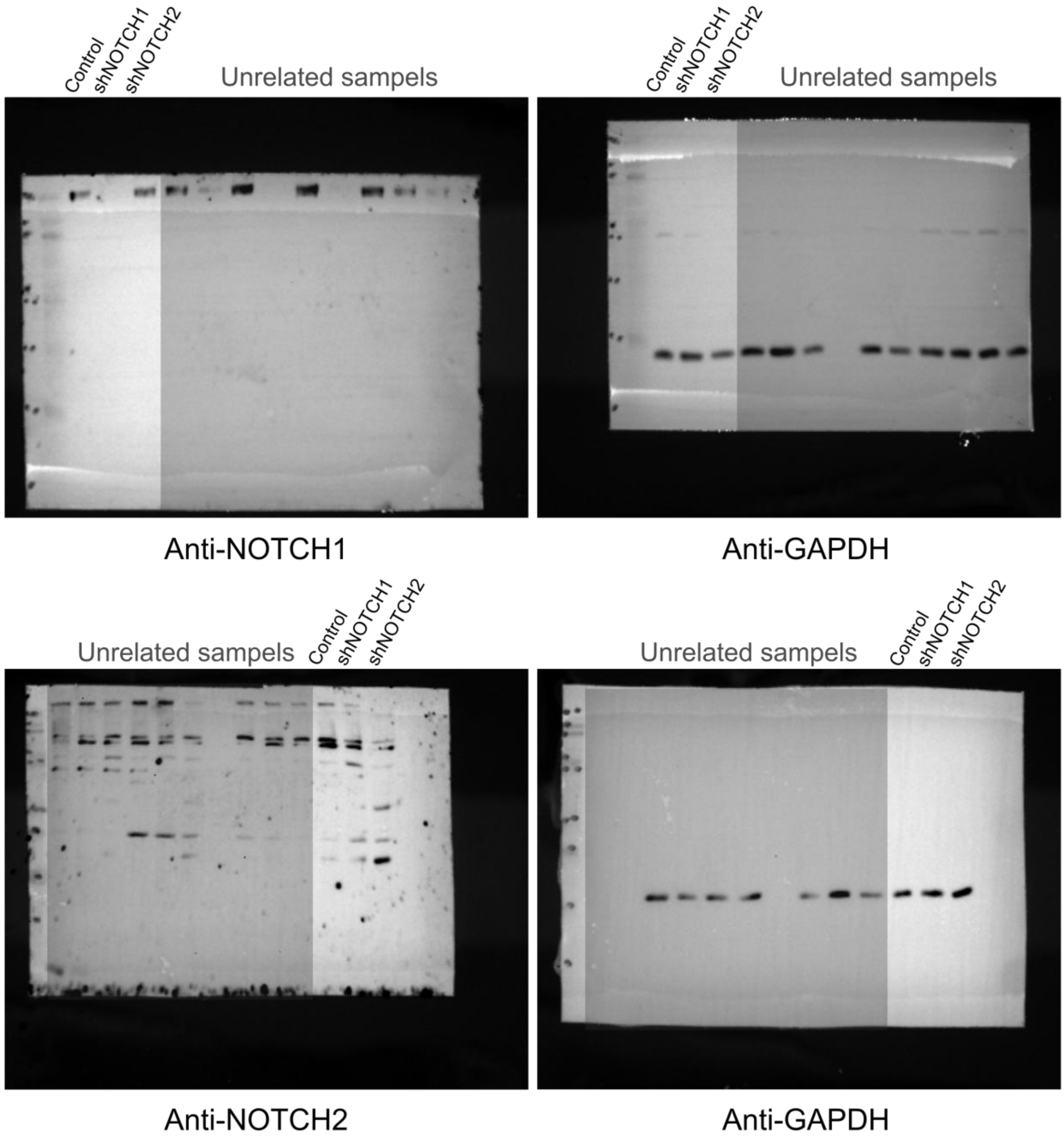

Figure S2. Unprocessed full-size Western blot membranes corresponding to the cropped regions shown in Figure 1b.
